# Development and Validation of Breastfeeding After Bariatric Surgery Scale

**DOI:** 10.1177/08903344261431944

**Published:** 2026-05-07

**Authors:** Seyedeh Samira Mokhlesi, Vidanka Vasilevski, Linda Sweet

**Affiliations:** 1 School of Nursing and Midwifery, Centre for Quality and Patient Safety Research in the Institute for Health Transformation, Deakin University, Burwood, VIC, Australia; 2 Western Health, St. Albans, VIC, Australia

**Keywords:** bariatric surgery, breastfeeding, factor analysis, lactation, psychometrics

## Abstract

**Background:**

With an increasing number of women of reproductive age undergoing bariatric surgery, understanding breastfeeding experiences in this population has become increasingly important. While several breastfeeding questionnaires exist, none have been specifically developed to capture the unique experiences and influencing factors relevant to women who have undergone bariatric surgery.

**Research Aim:**

The aim of this study was to develop and assess the psychometric properties of a breastfeeding questionnaire designed specifically for women with histories of bariatric surgery.

**Methods:**

The Breastfeeding after Bariatric Surgery Scale (BBSS) was developed and validated in three phases: Phase 1, generating items by reviewing the relevant literature and the theory of planned behaviour; Phase 2, evaluating content validity with a panel of experts; and Phase 3, evaluating face validity, exploratory factor analysis with varimax rotation, and reliability and responsiveness.

**Results:**

Initially, 40 items were generated in Phase 1. After assessing content and face validity, the number of items was reduced to 22. The results of exploratory factor analysis yielded a scale with 22 items in five domains, including “healthcare provider support,” “perceived behavioural control,” “subjective norms,” “positive breastfeeding experience,” and “negative feelings and challenges.” These subscales explained 63.19 and 69.70% of the total variance, respectively. Cronbach’s alpha coefficient for each subscale was >0.70, and the intraclass correlation coefficient for each subscale was >0.75.

**Conclusion:**

This scale showed acceptable validity and reliability for measuring breastfeeding behaviour and experiences in mothers who have had bariatric surgery.

## Background

In recent decades, global obesity rates have increased dramatically, particularly among women of reproductive age (Jelaković et al., 2023). Australia has one of the highest rates of overweight and obesity among high-income countries, affecting more than half of women of reproductive age in 2022 (Australian Bureau of Statistics, 2023). Obesity can impact all aspects of women’s reproductive health, including family planning, fertility, pregnancy, and sexual health (Albrecht et al., 2025).

Bariatric surgery is an effective treatment for people with severe obesity who cannot lose weight through traditional methods such as diet and exercise (Maxim et al., 2025). In 2023, up to 80% of individuals undergoing bariatric surgery in Australia were women of reproductive age (Bariatric Surgery Registry, 2024). A significant number of women with obesity and infertility consider bariatric surgery as a potential solution (Butterworth et al., 2016). Fertility is usually improved after bariatric surgery because weight loss can prompt the restoration of ovulation (Baharuddin et al., 2021; Dilday et al., 2017). With improved fertility rates and an increasing number of women of reproductive age undergoing bariatric surgery, supporting post–bariatric surgery mothers who desire to breastfeed is an important topic.

Breastfeeding provides significant health benefits for both mothers and infants, including reduced risks of metabolic and cardiovascular conditions for mothers and lower rates of infection and obesity in infants (Brahm & Valdes, 2017; Del Ciampo & Del Ciampo, 2018; Moubareck, 2021). Gimenes et al. (2018) reported that infants of post–bariatric surgery mothers who received exclusive breastfeeding were more likely to present with lower glycemia values throughout childhood and lower fat mass than those who received artificial formula or combination feeding.

Due to the significant benefits of breastfeeding, the World Health Organization (WHO) has set a global target for 60% of infants to be exclusively breastfed until 6 months of age by 2030 (WHO, 2025). Contrary to WHO recommendations, fewer than half of infants aged 0–6 months globally were exclusively breastfed over the period of 2015–20 (WHO, 2023). Despite recent clinical practice guidelines recommending breastfeeding after bariatric surgery (Suri et al., 2024), there is a tendency for lower breastfeeding rates and shorter durations in women with histories of bariatric surgery compared with those who have not (Mokhlesi et al., 2024). Breastfeeding after bariatric surgery may be affected by multiple factors, such as limited support and information (Vasilevski et al., 2023), excess skin from substantial weight loss (Wambach & Spencer, 2019), reduced prolactin levels (Wang et al., 2020), and worries about the nutritional adequacy of breast milk for the infant’s health (Sweet & Vasilevski, 2022). However, the exact reasons for the decreased rate of breastfeeding after bariatric surgery are unclear (Vasilevski et al., 2023). There is a need to conduct more research that may help to better understand breastfeeding experiences and the factors influencing breastfeeding among women who have undergone bariatric surgery (Mokhlesi et al., 2025). Use of a questionnaire is common to assess health-related experiences and behaviours (Marshall, 2005). A number of questionnaires have been developed to assess breastfeeding behaviour and factors that impact breastfeeding success (Čatipović et al., 2023). Although generic breastfeeding questionnaires are valid, they may not be suitable for measuring breastfeeding behaviours of specific populations, such as mothers after bariatric surgery (Streiner et al., 2024). Mothers with a history of bariatric surgery face unique breastfeeding challenges (Mokhlesi et al., 2026). Specific questionnaires address the unique experiences and challenges of specific groups, providing insights that generic questionnaires may overlook (Streiner et al., 2024). To our knowledge, there is no specific, valid, and reliable breastfeeding questionnaire specifically for mothers who have undergone bariatric surgery. Given the increasing prevalence of bariatric surgery and the importance of breastfeeding, the aim of this study was to develop and assess the psychometric properties of a breastfeeding questionnaire developed for mothers after bariatric surgery.

**Key Messages**

• With rising rates of bariatric surgery among women of reproductive age and improved fertility postoperatively, supporting breastfeeding in this population is increasingly important.

• Women who have undergone bariatric surgery often face unique breastfeeding challenges.

• The Breastfeeding After Bariatric Surgery Scale is the first psychometrically validated tool specifically designed to assess breastfeeding experiences and behaviours in mothers who have had bariatric surgery.

• The use of this valid and reliable, population-specific breastfeeding scale enables healthcare providers to assess the factors influencing breastfeeding practices, allowing for the development of tailored education and support strategies.

## Methods

**Figure 1.**
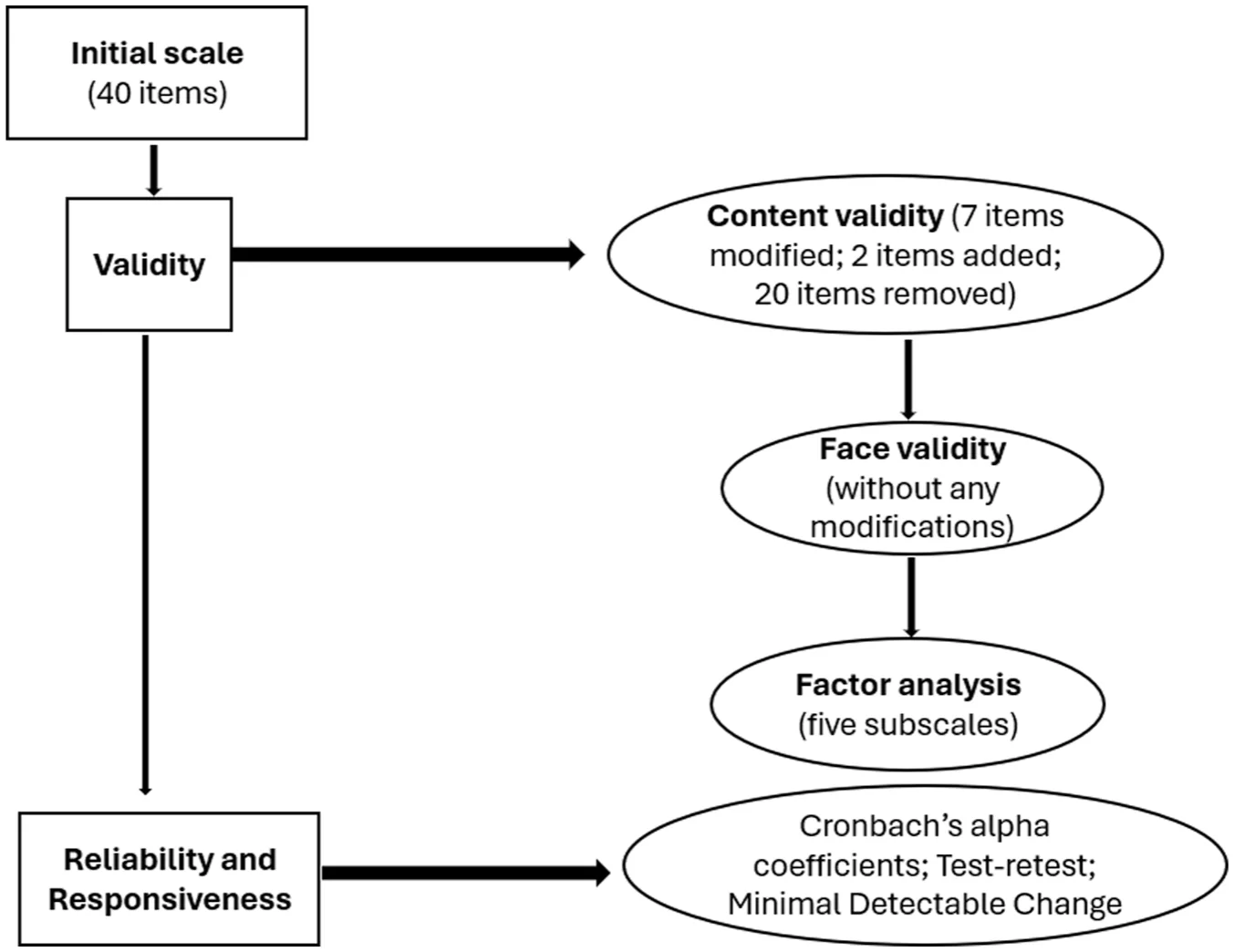
Process of development and psychometric analysis of the Breastfeeding After Bariatric Surgery Scale.

The process of instrument development and validation of items for the Breastfeeding After Bariatric Surgery Scale (BBSS) was undertaken in the following three phases: Phase 1, generating items by reviewing the relevant literature and the theory of planned behaviour (TPB) (Ajzen, 2011); Phase 2, evaluating content validity by sending the first draft of the scale to a panel of expert reviewers; and Phase 3, evaluating face validity, exploratory factor analysis with varimax rotation, and reliability and responsiveness. Figure 1 displays the process of developing the scale and conducting the psychometric analysis. A list of removed items, including the stage and reason for exclusion, is provided in Supplementary Table S1 in the online version of the journal. This study was approved by the Deakin University Human Research Ethical Committee (DUHREC 2024–243). Participation was voluntary and anonymous. Participants could withdraw at any time.

### Development of the BBSS

BBSS content was derived from two literature reviews and was guided by the TPB (Ajzen, 2011). The first literature review (Mokhlesi et al., 2024) ensured that the survey questions reflected the evidence regarding the breastfeeding experiences of mothers who have had bariatric surgery. The second literature review (under review) focused on existing breastfeeding questionnaires and supported the development and wording of questions regarding potential challenges and facilitators to breastfeeding in these mothers. The TPB was employed to guide the design of the breastfeeding scale, and each component of the TPB (i.e., attitudes, subjective norms, and perceived behavioural control) was included in its development (Ajzen, 2011). The decision to use the TPB as a guide to develop the BBSS was based on evidence indicating that maternal attitudes, subjective norms, and perceived behavioural control were significant predictors of breastfeeding intentions, even after controlling for maternal education and perceived insufficient milk production (Lau et al., 2018). The initial scale that was developed included 40 items. Of these, eight items were related to knowledge, seven items were focussed on subjective norms, three items reflected perceived behavioural control, five items were related to support for breastfeeding, and 11 items were about breastfeeding experience. All subscales were rated on a 5-point Likert scale (ranging from “Strongly agree” to “Strongly disagree”) except the subjective norms domain, which used a 6-point Likert scale (ranging from “Strongly agree” to “Strongly disagree” with an option for “Not applicable”).

### Content Validity

Both qualitative and quantitative approaches were used to assess the content validity of the scale. A panel of seven experts evaluated the qualitative content validity, and nine evaluated the quantitative content validity, aligning with the recommendation to involve five to 10 experts for content validity (Polit & Beck, 2020). This panel included dietitians specialising in maternal nutrition, midwives or nurses who specialise in lactation, and an expert in measurement and instrument design.

For the qualitative content validity assessment, we emailed the BBSS to the panel and asked panel members to provide feedback on features, including the length of the scale, how easy the scale was to follow, whether the scale items were easy to understand, whether the scoring method was appropriate, whether any questions might be considered offensive/should be removed, whether any new scale items should be included, and any other comments/suggestions for improving the scale. The feedback from the panel was collated and documented in a summary table. The research team discussed the comments and suggestions and made changes to the first draft of the scale accordingly.

In the next stage, the quantitative content validity of the revised scale was assessed by computing the content validity index (CVI) and content validity ratio (CVR) based on experts’ responses (DeVellis & Thorpe, 2021). The panel assessed the survey items for relevance, clarity, simplicity, and necessity. The research team contacted these experts via email. Interested experts were directed to an online structured evaluation form with a Likert scale set up in REDCap using the link provided via email. Experts were asked to evaluate the CVI by scoring each item’s simplicity, relevance, and clarity using a 4-point Likert scale, where 1 indicates “Not simple, not relevant, and not clear” and 4 indicates “Very simple, very relevant, and very clear” (Polit & Beck, 2020). In addition, the CVR was used to evaluate the necessity of each item. Experts rated the items on a 3-point scale: 1 for “Not necessary,” 2 for “Useful but not essential,” and 3 for “Essential” (DeVellis & Thorpe, 2021; Polit & Beck, 2020). Item-level CVR (I-CVR) was calculated using Lawshe’s formula, which compares the number of experts who rated the item as essential with the total number of experts (Lawshe, 1975; Polit & Beck, 2020). According to Lawshe’s table, an acceptable I-CVR value for nine experts is 0.78 (Lawshe, 1975; Polit & Beck, 2020). Item-level CVI (I-CVI) was calculated by dividing the number of experts who rated each item as 3 or 4 by the total number of experts (Almanasreh et al., 2019; Polit & Beck, 2020). An item was considered valid if its I-CVI was ≥0.79 (Polit & Beck, 2020). The modified kappa (κ^*^) was calculated to adjust the I-CVI for chance agreement using the formula κ^*^ = I-CVI − *P_c_*/1 − *P_c_* (Polit & Beck, 2020), where *P_c_* represents the probability of chance agreement, calculated as *P_c_* = *N*!/*A*!(*N* − *A*)! × 0.5^N^ (where *N* is the number of experts, and *A* is the number of experts who rated the item as relevant) (Polit & Beck, 2020). A κ^*^ value >0.74 is excellent (Polit & Beck, 2020). Scale-level CVI (S-CVI) was computed by calculating the mean of I-CVI values, where S-CVI >0.9% is considered acceptable (Almanasreh et al., 2019; Polit & Beck, 2020).

### Face Validity, Structural Validity, Reliability, and Responsiveness

#### Setting and Participants

Mothers who had undergone bariatric surgery before pregnancy were invited to participate in the study through advertisements posted on social media platforms (e.g., Facebook and Instagram). Participants were directed to the online scale in REDCap from the link provided in the social media advertisements. Eligible participants were women aged 18–45 years who had undergone bariatric surgery before pregnancy, experienced a singleton pregnancy, and had given birth to their most recent baby within the past 5 years. Studies have shown that women can accurately recall their breastfeeding practices for a long time postpartum (Li et al., 2020; Natland et al., 2012), supporting the inclusion of women who had their most recent baby within the last 5 years. Exclusion criteria included self-reported conditions that interfere with breastfeeding, such as mothers having had breast surgery, having infants with prematurity or cleft palate, or the necessity for medications that are incompatible with breastfeeding.

To enhance the authenticity of responses, eligibility screening questions were included at the beginning of the survey to ensure that participants met the inclusion criteria. Survey commencement was not permitted until all eligibility and consent statements were endorsed. The survey was administered via REDCap, which restricted submissions to one response per device and prevented multiple entries from the same participant. Participants were offered the opportunity to enter a prize draw to win one of two 50 AUD gift cards.

#### Face validity

Based on the advice of Polit and Beck (2020) to determine face validity of the BBSS, five mothers who met the inclusion criteria were asked to evaluate the items regarding their level of difficulty and ambiguity. Feedback about the scale items was gathered by including open-text boxes after each subset of items. A final open-text box asked women to provide feedback about usability, length, technical issues, and overall feedback. The feedback was collected through REDCap.

#### Structural validity

Exploratory factor analysis was conducted to assess the structural validity (Polit & Beck, 2020). Exploratory factor analysis was conducted to identify underlying concepts measured by the scale items (Polit & Beck, 2020). Since the breastfeeding experience subscale only applies to mothers who have breastfed, a sub-analysis was conducted using data exclusively from those mothers (whether they practised exclusive breastfeeding or mixed feeding). The other subscales (e.g., knowledge, subjective norms, perceived behavioural control, and support) were analysed using the entire sample because those items were relevant to all mothers regardless of infant feeding method. This approach was used to avoid inclusion of nonapplicable responses, which may distort factor structure and validity (DeVellis & Thorpe, 2021). In factor analysis, the sample size is often determined based on ratios such as 3:1 or 5:1 between variables and factors or by arbitrary numbers such as 100 or 200 participants (Watkins, 2018). Accordingly, 221 mothers who met the inclusion criteria completed the scale, of whom 211 reported having breastfed and were included in the sub-analysis of the breastfeeding experience subscale. The Kaiser-Meyer-Olkin (KMO) test and Bartlett’s test of sphericity were used to assess sample adequacy (Shrestha, 2021; Williams et al., 2010). KMO has a value between 0 and 1, with higher values indicating better sample adequacy (Shrestha, 2021). Also, if the KMO test and Bartlett’s test of sphericity are significant, the sample is considered adequate (Shrestha, 2021; Williams et al., 2010). An eigenvalue ≥1 and scree plot were used to determine the factors extracted (Kaiser, 1960; Williams et al., 2010). The cutoff value for the minimum acceptable factor loading was considered to be 0.35 based on the following formula CV = 5.152/ $\sqrt{\left(n-2\right)}$ (Hair et al., 2010). Finally, items with communality of >0.3, indicating poor shared variance with other items, were removed from the analysis (Polit & Beck, 2020).

### Reliability and Responsiveness

The internal consistency and test–retest methods were used to ensure the reliability of the scale (Polit & Beck, 2020). The internal consistency of the scale was evaluated using Cronbach’s alpha coefficients based on the same sample used for the construct validity analysis. A value >0.7 was regarded as acceptable (DeVellis & Thorpe, 2021; Polit & Beck, 2020). Instrument stability was assessed by the test–retest method and through completion of the scale by 11 mothers who met the inclusion criteria within a 2-week interval (DeVellis & Thorpe, 2021; Polit & Beck, 2020). Interested participants were directed to click a link in the advertisement to submit their email address. The student researcher sent an email with the link to the survey in REDCap. To evaluate the reliability of the questionnaire, these participants were emailed the survey link after 2 weeks to repeat the survey. Intraclass correlation (ICC) was used to assess reliability (DeVellis & Thorpe, 2021; Polit & Beck, 2020). If the ICC was >0.75, the stability was considered desirable (DeVellis & Thorpe, 2021; Polit & Beck, 2020).

*Responsiveness* refers to the ability of a scale to detect change over time in the construct to be measured (Mokkink et al., 2018). To determine the responsiveness of the scale, the minimal detectable change (MDC) was calculated (Seamon et al., 2022). First, the standard error of measurement (SEM) was determined using the formula: SEM = SD $\sqrt{1-\mathrm{ICC}}$ , where SD represents the standard deviation of the total score (Seamon et al., 2022). Then the MDC at a 95% confidence level was calculated using the following formula: MDC = SEM × $\sqrt{2}\times 1.96$ (Seamon et al., 2022). An MDC of <30% was considered acceptable (Mokkink et al., 2018).

## Results

### Content Validity

Based on the feedback provided by the panel of experts during the qualitative content validity assessment, several modifications were made to enhance the clarity and comprehensibility of the scale items. Table 1 provides examples of the modified, added, and deleted items based on expert feedback. The wording of seven items was modified to improve clarity and comprehensibility. Two items were added to address gaps identified by the experts. Eight items were removed because they were considered duplicates or were deemed unrelated to the study objectives. As a result of these adjustments, the initial 40 items were reduced to 34 items in the final first version of the scale.

**Table 1.** Examples of Item Modifications Based on Expert Feedback.

Action	Original item	Revised/new item
Modified	I trusted that my body could produce enough milk for my baby after bariatric surgery.	I trusted that my body could produce enough breastmilk to meet my baby’s feeding needs after bariatric surgery.
Modified	I received specific nutritional guidance from my healthcare worker related to breastfeeding after bariatric surgery.	I received specific nutritional guidance from my healthcare worker related to what to eat and drink if I chose to breastfeed after bariatric surgery.
Added	N/A	After bariatric surgery, weight management is difficult for breastfeeding women.
Added	N/A	I received specific advice about what supplements to take if I chose to breastfeed after bariatric surgery.
Deleted	After my baby’s birth, a healthcare worker assisted me with breastfeeding after bariatric surgery.	N/A
Deleted	I could manage to keep up with my baby’s breastfeeding demands after bariatric surgery.	N/A

In the quantitative content analysis, the CVR, CVI, and modified kappa (κ^*^) were calculated for each item. Based on Lawshe’s cutoff point, 12 items with a CVR <0.75 were removed, reducing the total number of items from 34 to 22. All items had a CVI >0.79 and a κ^*^ >0.74, so none were removed at this stage. The S-CVI was calculated as 0.97.

### Face Validity

Demographic information about the mothers (*n* = 5) who participated in the face validity component of the study is shown in Table 2. Most participants were aged 30–34 years and held postgraduate qualifications (*n* = 3; 60%). Sleeve gastrectomy was the most common type of surgery (*n* = 4; 80%), and all five participants reported losing 30 kg or more following bariatric surgery. All participants agreed that the items were clearly written and at an appropriate difficulty level. As a result, all 22 items were retained without any modifications.

**Table 2.** Demographic Characteristics of Mothers Who Participated in Psychometric Testing Steps.

Characteristic	Face validity (*n* = 5), *n* (%)	Factor analysis and internal consistency		Test–retest (*n* = 11), *n* (%)
		Main sample (*n* = 221), *n* (%)	Breastfeeding experience subscale^a^ (*n* = 211), *n* (%)	
Age (years)				
18–24	0	1 (0.4)	1 (0.5)	0
25–29	1 (20)	26 (11.8)	24 (11.4)	2 (18.2)
30–34	3 (60)	87 (39.4)	84 (39.8)	4 (36.4)
35–39	1 (20)	70 (31.7)	65 (30.8)	2 (18.2)
40–45	0	37 (16.7)	37 (17.5)	3 (27.2)
Education				
Postgraduate	3 (60)	44 (20.1)	44 (20.9)	3 (27.2)
Bachelor	0	55 (25.1)	53 (25.1)	2 (18.2)
Diploma	2 (40)	75 (34.3)	74 (35)	5 (45.5)
High school	0	43 (19.6)	38 (18)	1 (9.1)
Prefer not to say	0	2 (0.9)	2 (0.9)	0
Type of bariatric surgery				
Sleeve gastrectomy	4 (80)	166 (75.5)	158 (74.9)	9 (81.8)
Roux-en-Y gastric bypass	1 (20)	32 (14.5)	32 (15.2)	1 (9.1)
One-anastomosis gastric bypass	0	7 (3.2)	7 (3.3)	0
Adjusted gastric band	0	6 (2.7)	6 (2.8)	0
Other	0	1 (0.5)	1 (0.5)	1 (9.1)
Multi	0	8 (3.6)	7 (3.3)	0
Time between bariatric surgery and recent pregnancy (months)				
<6	0	6 (2.7)	6 (2.8)	0
6–11	1 (20)	25 (11.3)	25 (11.8)	2 (18.2)
12–17	2 (40)	26 (11.8)	26 (12.3)	5 (45.5)
18–23	0	26 (11.8)	26 (12.3)	0
≥24	2 (40)	138 (62.4)	128 (60.7)	4 (36.3)
Weight loss before recent pregnancy				
Gained weight	0	5 (2.3)	4 (1.9)	0
No change in weight	0	3 (1.4)	3 (1.4)	0
1–9 kg	0	2 (0.9)	2 (0.9)	0
10–19 kg	0	10 (4.6)	10 (4.7)	1 (9.1)
20–29 kg	0	31 (14.2)	30 (14.2)	1 (9.1)
≥30 kg	5 (100)	167 (76.6)	162 (76.7)	9 (81.8)
Parity				
Primiparous	0	103 (46.6)	98 (46.4)	5 (45.5)
Infant age at survey completion (months)				
<6	0	69 (31.2)	67 (31.8)	1 (9.1)
6–12	2 (40)	61 (27.6)	59 (28)	6 (54.5)
13–24	1 (20)	44 (19.9)	40 (19)	2 (18.2)
24–36	1 (20)	27 (12.2)	25 (11.8)	2 (18.2)
>36	1 (20)	20 (9.1)	20 (9.5)	0
Mode of infant feeding during first 3 months				
Breastfeeding	5 (100)	67 (30.3)	67 (31.8)	4 (36.3)
Expressed milk	0	2 (0.9)	2 (0.9)	0
Both breastfeeding and expressed milk	0	36 (16.3)	36 (17.1)	2 (18.2)
Formula feeding	0	10 (4.5)	0	0
Mixed feeding (breastmilk and formula)	0	106 (48)	106 (50.2)	5 (45.5)

a The breastfeeding experience subscale represents a subset of the main sample and includes only participants who reported any breastfeeding (exclusive or mixed feeding).

### Structural Validity

Demographic information about participants is shown in Table 2. Exploratory factor analysis was conducted on the full sample of mothers (*N* = 221) for subscales assessing knowledge (two items), subjective norms (three items), perceived behavioural control (six items), and support (four items). The value of 0.79 obtained from the KMO test and the significance of Bartlett’s test result (*df* = 105, *χ*^2^ = 1,666.755, *P* < 0.001) suggested that the sampling was adequate. The initial analysis revealed four factors with eigenvalues >1, explaining 70.78% of the total variance. However, based on theoretical considerations, three factors were considered, accounting for 63.19% of the total variance (Table 3). All items had factor loadings of ≥0.35; therefore, 15 items were retained. Subsequently, each factor was labelled based on the content of its items. The three factors in the BBSS included healthcare provider support (seven items), perceived behavioural control (five items), and subjective norms (three items).

**Table 3.** Exploratory Factor Analysis Results for the Knowledge, Subjective Norms, Perceived Behavioural Control, and Support Subscales (*n* = 221).

Factor	Item	Factor loading	Communality	Eigenvalue	Variance (%)
Healthcare provider support	Q20. I received specific nutritional guidance from my healthcare worker related to what to eat and drink if I chose to breastfeed after bariatric surgery.	0.841	0.713	4.34	28.94
	Q21. I received specific advice about what supplements to take if I chose to breastfeed after bariatric surgery.	0.836	0.700		
	Q22. I received specific guidance from my healthcare worker on how to manage breastfeeding challenges related to my bariatric surgery.	0.834	0.696		
	Q19. Before my baby’s birth, a healthcare worker gave me breastfeeding advice related to bariatric surgery.	0.779	0.613		
	Q13. I was able to find the information I needed about breastfeeding challenges after bariatric surgery.	0.615	0.500		
	Q14. I knew who to ask if I had any questions about breastfeeding after bariatric surgery.	0.597	0.439		
	Q15. I sought advice from my healthcare provider about breastfeeding after bariatric surgery.	0.440	0.319		
Perceived behavioural control	Q10. I trusted that my body could produce enough breastmilk to meet my baby’s feeding needs after bariatric surgery.	0.894	0.800	3.24	21.63
	Q12. I was confident that I would be able to breastfeed my baby so that they would gain enough weight after bariatric surgery.	0.878	0.792		
	Q9. I was confident that I could breastfeed despite my bariatric surgery.	0.832	0.710		
	Q3. After bariatric surgery, breast milk contains all the nutrients a baby needs for the first 6 months.	0.651	0.456		
	Q1. Women who have had bariatric surgery can successfully breastfeed.	0.611	0.418		
Subjective norms	Q18. My closest friend(s) thought I should breastfeed even though I had bariatric surgery.	0.900	0.833	1.89	12.62
	Q16. My partner thought I should breastfeed even though I had bariatric surgery.	0.867	0.790		
	Q17. My parents thought I should breastfeed even though I had bariatric surgery.	0.813	0.705		

For the breastfeeding experience subscale (*n* = 211), sampling adequacy was confirmed with a KMO test value of 0.74, and Bartlett’s test of sphericity was significant (*χ*^2^ = 723.382, *df* = 21, *P* < 0.001). The initial analysis identified two factors with eigenvalues >1, which together explained 69.70% of the total variance. These two factors were retained based on the scree plot and theoretical considerations (Table 4). All items had factor loadings of ≥0.35; therefore, seven items were retained. Subsequently, each factor was labelled based on the content of its items. The two factors included positive breastfeeding experiences (four items) and negative feelings and challenges (three items).

**Table 4.** Exploratory Factor Analysis for the Breastfeeding Experience Subscale (*n* = 211).

Factor	Item	Factor loading	Communality	Eigenvalue	Variance (%)
Positive breastfeeding experiences	Q27. The fact that I could produce breastmilk to feed my own baby after bariatric surgery was very satisfying.	0.919	0.850	3.33	47.62
	Q26. Breastfeeding after bariatric surgery made me feel more confident as a mother.	0.889	0.804		
	Q29. My baby gained weight well with my breastmilk after my bariatric surgery	0.870	0.770		
	Q28. While breastfeeding after bariatric surgery, I did not worry about my baby gaining enough weight.	0.576	0.437		
Negative feelings and challenges	Q32. I was anxious to have my body back while breastfeeding after bariatric surgery.	0.860	0.752	1.54	22.07
	Q33. Breastfeeding after bariatric surgery made it difficult for me to manage my weight.	0.782	0613		
	Q30. It was a burden being my baby’s main source of food after bariatric surgery	0.747	0.657		

### Reliability and Responsiveness

The internal consistency of the scale was evaluated using Cronbach’s coefficient alpha. The coefficient ranged from 0.75 to 0.86 across the subscales, indicating proper internal consistency. Tool stability was tested by a test–retest method via the ICC. The ICC ranged from 0.89 to 0.94 for the subscales, demonstrating acceptable stability of the scale (Table 5). The SEM was ±6.90, and the MDC at the 95% confidence level was 19.1 points.

**Table 5.** Reliability Measures of the Breastfeeding After Bariatric Surgery Scale.

Subscale	Cronbach’s alpha	Intraclass correlation (95% confidence interval)
Healthcare provider support	0.81	0.94 (0.89–0.98)
Perceived behavioural control	0.79	0.92 (0.84–0.97)
Subjective norms	0.86	0.90 (0.77–0.96)
Positive breastfeeding experience	0.81	0.89 (0.76–0.96)
Negative feelings and challenges	0.75	0.92 (0.84–0.97)

### Scoring Procedure

The final BBSS consists of five subscales with a total of 22 questions. All subscales were rated on a 5-point Likert scale (ranging from “Strongly agree” = 5 to “Strongly disagree” = 1) except for the subjective norms subscale, which used a 6-point Likert scale (ranging from “Strongly agree” = 5 to “Strongly disagree” = 1 with an option for “Not applicable” = 0). The positive breastfeeding experiences, negative feelings, and challenges subscales only apply to mothers who have breastfed. The items relating to the subscale “Negative feelings and challenges” were reverse scored, where the answer “Strongly agree” was scored as 1 and the answer “Strongly disagree” was scored as 5.

## Discussion

This is the first study to develop and validate a reliable scale to assess the breastfeeding behaviours and experiences of mothers who have bariatric surgery prior to pregnancy. The BBSS consists of 22 items within five subscales: “Healthcare provider support,” “Perceived behavioural control,” and “Subjective norms” (for all mothers) as well as “Positive breastfeeding experience” and “Negative feelings and challenges” (for mothers who have breastfed). These subscales explained 63.19 and 69.70% of the total variance, respectively. The acceptable explained variance supports the scale’s ability to measure the concept of breastfeeding behaviours and experiences among women who have prepregnancy bariatric surgery (Hair et al., 2010).

The first factor extracted in the factor analysis was healthcare provider support. This factor had the highest explained variance and is considered a key dimension of the scale. Breastfeeding support provided by healthcare professionals can be done in several ways. Informational support by healthcare providers is one approach for providing guidance and advice to breastfeeding mothers (Blixt et al., 2019). Women who have had bariatric surgery require tailored advice during pregnancy and breastfeeding, but they often receive limited individualised care throughout these times (Vasilevski et al., 2023). Because bariatric surgeries often alter nutrient intake capacity and absorption, specific nutritional guidance and supplementation advice are needed for women with histories of bariatric surgery (Walędziak et al., 2021). In three qualitative studies, participants emphasised the importance of receiving specific and tailored breastfeeding information to address their unique needs after bariatric surgery (Crill et al., 2009; Hendrix et al., 2011; Vasilevski et al., 2023). Without appropriate breastfeeding information, mothers may feel less confident to start or continue breastfeeding (James et al., 2020).

The second factor extracted in the factor analysis was perceived behavioural control. Perceived behavioural control and self-efficacy are quite similar, and both are related to perceived ability and confidence to perform a behaviour (Ajzen, 2002). Maternal breastfeeding self-efficacy is defined as a mother’s confidence in her ability to successfully breastfeed her infant (De Roza et al., 2019). Although not explicitly studied in the context of bariatric surgery, maternal confidence in breastfeeding was shown in some studies to be positively associated with intention, duration, and exclusivity of breastfeeding (Clapton-Caputo et al., 2021; James et al., 2020). The most frequent factor load in the “Perceived behavioural control” subscale was “I trusted that my body could produce enough breastmilk to meet my baby’s feeding needs after bariatric surgery,” indicating that confidence in milk supply is a key part of breastfeeding for women after bariatric surgery. Maternal concern about milk supply is the most common reason for stopping breastfeeding (Awaliyah et al., 2019; De Roza et al., 2019). Milk supply can be more challenging for mothers having undergone bariatric surgery because reduced stomach capacity and psychological factors may limit food intake and daily caloric consumption during breastfeeding (Mahan & Raymond, 2016). Furthermore, prolactin levels drop within a year after bariatric surgery, possibly affecting breastmilk supply and breastfeeding success (Wang et al., 2020). A qualitative study showed that many mothers who had bariatric surgery chose formula feeding because of a perceived lack of milk supply (Crill et al., 2009). Breastfeeding confidence is shaped not only by a woman’s own experiences but also by the views and support of others, which can either boost or reduce her belief in her ability to breastfeed (Lau et al., 2018).

The third factor extracted in the factor analysis was subjective norms. The *subjective norms* of breastfeeding refer to a mother’s perception of encouragement and support from her social network (Zhang et al., 2018). Based on the TPB, subjective norms are considered an important factor in breastfeeding behaviour (Guo et al., 2016). The degree to which breastfeeding is accepted or rejected by significant others, such as family members, partners, or friends, can significantly influence a mother’s decision to breastfeed (Rostamkhan et al., 2020). Research has shown that breastfeeding mothers rely on practical and/or emotional support from their partners, family members, and peers (Chang et al., 2022). Such support is important to sustain breastfeeding, particularly when the mother is experiencing difficulties, and it can lead to a more positive breastfeeding experience (Guyer et al., 2012).

The final two factors identified in the BBSS were positive breastfeeding experience and negative feelings and challenges, both relating only to mothers who have breastfed after bariatric surgery. Most items in the “Positive breastfeeding experience” subscale focused on milk supply and the baby’s weight gain. Given that mothers who have undergone bariatric surgery often perceive themselves to have insufficient breastmilk production (Crill et al., 2009), experiencing adequate milk supply and healthy infant weight gain may be particularly satisfying for them. In contrast, the most frequent factor load in the “Negative feelings and challenges” subscale was related to maternal weight management and body-image concerns among mothers who breastfed after bariatric surgery. Because weight loss is the main goal of bariatric surgery, women who have had this surgery may face extra challenges during the postpartum period, especially with changes in their body weight and eating habits while breastfeeding (Suri et al., 2024). Postpartum body weight and body image can influence breastfeeding experiences and outcomes (Kapa et al., 2022). Although this issue was not directly studied in women after bariatric surgery, one study found that worries about weight gain and body image were linked to shorter breastfeeding duration (Brown et al., 2015). Greater concerns about weight gain and body image during pregnancy and after birth were associated with stopping breastfeeding earlier (Brown et al., 2015).

### Implications for Research and Practice

The BBSS provides a research tool to examine breastfeeding behaviours, experiences, and influencing factors among women with a history of bariatric surgery. The scale can be used in future studies to identify key barriers and facilitators to breastfeeding, evaluate associations with breastfeeding outcomes, and assess the impact of targeted interventions. In practice-oriented research, the BBSS may inform the development and evaluation of tailored educational resources and support strategies for this population as well as guide training priorities for healthcare providers involved in post–bariatric surgery maternity care.

### Strengths and Limitations

To our knowledge, this is the first breastfeeding scale specifically for mothers who have undergone bariatric surgery prior to pregnancy. A key strength of the study is the diversity of the participant group, which included variations in age, education level, parity, type of bariatric surgery, amount of weight loss after surgery, the time interval between surgery and pregnancy, and infant feeding methods. One limitation of this study is the relatively small sample size used for the test–retest reliability assessment (*n* = 11), which may limit the precision of the stability estimates. Also, the questionnaire was developed in Australia, and it may be limited in generalisability to similar contexts. Therefore, it is recommended that the psychometric properties of the BBSS be evaluated in different countries and settings. Additionally, to further ensure the scale’s robustness, future research should assess its psychometric properties using different statistical approaches, such as confirmatory factor analysis.

## Conclusion

The BBSS is a simple, valid, and reliable scale for assessing the breastfeeding behaviour and experiences of women who have had bariatric surgery before pregnancy. It addresses a gap in existing breastfeeding assessment tools by focusing on the unique experiences of this group of women. This scale provides researchers with a valid and reliable tool to study breastfeeding outcomes in this underresearched population.

## Supplemental Material

sj-docx-1-jhl-10.1177_08903344261431944 – Supplemental material for Development and Validation of Breastfeeding After Bariatric Surgery Scale Supplemental material, sj-docx-1-jhl-10.1177_08903344261431944 for Development and Validation of Breastfeeding After Bariatric Surgery Scale by Seyedeh Samira Mokhlesi, Vidanka Vasilevski and Linda Sweet in Journal of Human Lactation
