# Supplementary material for: Development and Validation of Breastfeeding After Bariatric Surgery Scale
Source: J Hum Lact. 2026 May 7;42(3):795–806. doi: 10.1177/08903344261431944 (PMC13615319; doi:10.1177/08903344261431944)
Supplement: Supplementary file 1 [file sj-docx-1-jhl-10.1177_08903344261431944.docx]

Supplementary Table S1. Item reduction process for the Breastfeeding after Bariatric Surgery Scale (BBSS)

|  | Item content | Stage removed | Reason for exclusion |
| --- | --- | --- | --- |
| 1 | After bariatric surgery, exclusive breastfeeding (no water, other food or solids) is recommended for the first 6 months. | Quantitative Content validity | CVR < 0.75 |
| 2 | After bariatric surgery, women who breastfeed need additional calories. | Quantitative Content validity | CVR < 0.75 |
| 3 | After bariatric surgery, women who breastfeed need additional protein. | Quantitative Content validity | CVR < 0.75 |
| 4 | After bariatric surgery, women who breastfeed need additional multivitamin and mineral supplementation. | Quantitative Content validity | CVR < 0.75 |
| 5 | After bariatric surgery, the growth of breastfed infants is impacted by the mother's caloric intake and nutritional quality. | Quantitative Content validity | CVR < 0.75 |
| 6 | After bariatric surgery, weight management is difficult for breastfeeding women. | Quantitative Content validity | CVR < 0.75 |
| 7 | I was confident that I would be able to determine if my baby was getting enough breastmilk | Quantitative Content validity | CVR < 0.75 |
| 8 | There were things I wanted to know about breastfeeding after bariatric surgery that I was not told. | Quantitative Content validity | CVR < 0.75 |
| 9 | I felt a sense of inner contentment with breastfeeding after bariatric surgery. | Quantitative Content validity | CVR < 0.75 |
| 10 | Breastfeeding after bariatric surgery was a very nurturing, maternal experience. | Quantitative Content validity | CVR < 0.75 |
| 11 | Breastfeeding after bariatric surgery was physically draining. | Quantitative Content validity | CVR < 0.75 |
| 12 | Breastfeeding after bariatric surgery had a positive impact on my personal wellbeing | Quantitative Content validity | CVR < 0.75 |
| 13 | I was physically able to breastfeed after bariatric surgery. | Qualitative Content validity | duplicates or were deemed unrelated |
| 14 | I needed help to breastfeed because of my bariatric surgery. | Qualitative Content validity | duplicates or were deemed unrelated |
| 15 | I could manage to keep up with my baby’s breastfeeding demands after bariatric surgery. | Qualitative Content validity | duplicates or were deemed unrelated |
| 16 | I could talk to my healthcare provider about breastfeeding my baby after bariatric surgery | Qualitative Content validity | duplicates or were deemed unrelated |
| 17 | I accepted advice from my healthcare provider about breastfeeding after bariatric surgery. | Qualitative Content validity | duplicates or were deemed unrelated |
| 18 | After my baby’s birth, a healthcare worker assisted me with breastfeeding after bariatric surgery. | Qualitative Content validity | duplicates or were deemed unrelated |
| 19 | If necessary, there was someone other than family or friends (e.g. health care provider, breastfeeding support group member) whom I could consult on the unique breastfeeding challenges post-bariatric surgery. | Qualitative Content validity | duplicates or were deemed unrelated |
| 20 | There was someone close to me who was able to give me emotional support whilst breastfeeding. | Qualitative Content validity | duplicates or were deemed unrelated |
